# Studying the rapid bioconversion of lignocellulosic sugars into ethanol using high cell density fermentations with cell recycle

**DOI:** 10.1186/1754-6834-7-73

**Published:** 2014-05-15

**Authors:** Cory Sarks, Mingjie Jin, Trey K Sato, Venkatesh Balan, Bruce E Dale

**Affiliations:** 1 Biomass Conversion Research Laboratory (BCRL), Department of Chemical Engineering and Materials Science, Michigan State University, 3815 Technology Boulevard, Lansing, MI 48910, USA; 2 DOE Great Lakes Bioenergy Research Center (GLBRC), Michigan State University, East Lansing, MI 48824, USA; 3 DOE Great Lakes Bioenergy Research Center, University of Wisconsin-Madison, 1552 University Avenue, Madison, WI 53726, USA

**Keywords:** Lignocellulosic biofuel, Ethanol fermentation, *Saccharomyces cerevisiae*, AFEX, Cell recycling, RaBIT

## Abstract

**Background:**

The Rapid Bioconversion with Integrated recycle Technology (RaBIT) process reduces capital costs, processing times, and biocatalyst cost for biochemical conversion of cellulosic biomass to biofuels by reducing total bioprocessing time (enzymatic hydrolysis plus fermentation) to 48 h, increasing biofuel productivity (g/L/h) twofold, and recycling biocatalysts (enzymes and microbes) to the next cycle. To achieve these results, RaBIT utilizes 24-h high cell density fermentations along with cell recycling to solve the slow/incomplete xylose fermentation issue, which is critical for lignocellulosic biofuel fermentations. Previous studies utilizing similar fermentation conditions showed a decrease in xylose consumption when recycling cells into the next fermentation cycle. Eliminating this decrease is critical for RaBIT process effectiveness for high cycle counts.

**Results:**

Nine different engineered microbial strains (including *Saccharomyces cerevisiae* strains, *Scheffersomyces (Pichia) stipitis* strains, *Zymomonas mobilis* 8b, and *Escherichia coli* KO11) were tested under RaBIT platform fermentations to determine their suitability for this platform. Fermentation conditions were then optimized for *S. cerevisiae* GLBRCY128. Three different nutrient sources (corn steep liquor, yeast extract, and wheat germ) were evaluated to improve xylose consumption by recycled cells. Capacitance readings were used to accurately measure viable cell mass profiles over five cycles.

**Conclusion:**

The results showed that not all strains are capable of effectively performing the RaBIT process. Acceptable performance is largely correlated to the specific xylose consumption rate. Corn steep liquor was found to reduce the deleterious impacts of cell recycle and improve specific xylose consumption rates. The viable cell mass profiles indicated that reduction in specific xylose consumption rate, not a drop in viable cell mass, was the main cause for decreasing xylose consumption.

## Background

Biofuels have recently gained momentum in academic research, government, and large companies [1,2]. The benefits of biofuels have been widely accepted by society, although some controversy remains because of perceived conflicts with food production [3]. Nevertheless, biofuels represent a renewable option to replace a depleting oil supply and can help mitigate the climate change impacts of fossil fuel use [4]. Furthermore, a large-scale biofuel industry would improve energy security and strengthen the world economy.

Lignocellulosic biomass is a potential feedstock for low cost biofuel production [5]. The cost of lignocellulosic biomass is projected to be lower than prices for starch, which is currently used to produce bioethanol in the US [6,7]. Currently, agricultural residues such as corn stover (the leaves, husks, and stalks of corn plants) are usually left in fields [8]. There is also the potential to plant double crops and cover crops for added biomass productivity, improved soil chemistry, and fertilizer sequestration [9]. Additional biomass could be produced by cultivating dedicated energy crops on marginal lands unsuitable for food production [10].

Lignocellulosic biomass is highly recalcitrant and difficult to convert into monomeric sugars that can be fermented into biofuels by microorganisms [11]. Chemical and physical pretreatment processes are thus used to disrupt biomass allowing the structural carbohydrates to be more easily hydrolyzed to monomeric sugars by biomass degrading enzymes [11,12]. Thermochemical pretreatment processes generate degradation products that often are toxic to the microorganisms and reduce ethanol production and xylose consumption [13-15]. The high price of lignocellulosic enzymes and the long process times required for enzymatic hydrolysis and microbial fermentation are two bottlenecks preventing economical production of lignocellulosic ethanol [6]. To help overcome these bottlenecks, the Rapid Bioconversion with Integrated recycle Technology (RaBIT) process was developed in our laboratory. In previous publications and poster presentations we have referred to this process as BCRL SHF, BCRL SSCF, Fast SHF, and Fast SSCF (BCRL: Biomass Conversion Research Laboratory, SHF: separate hydrolysis and fermentation, SSCF: simultaneious saccharification and co-fermentation) [16].

**Figure 1:**
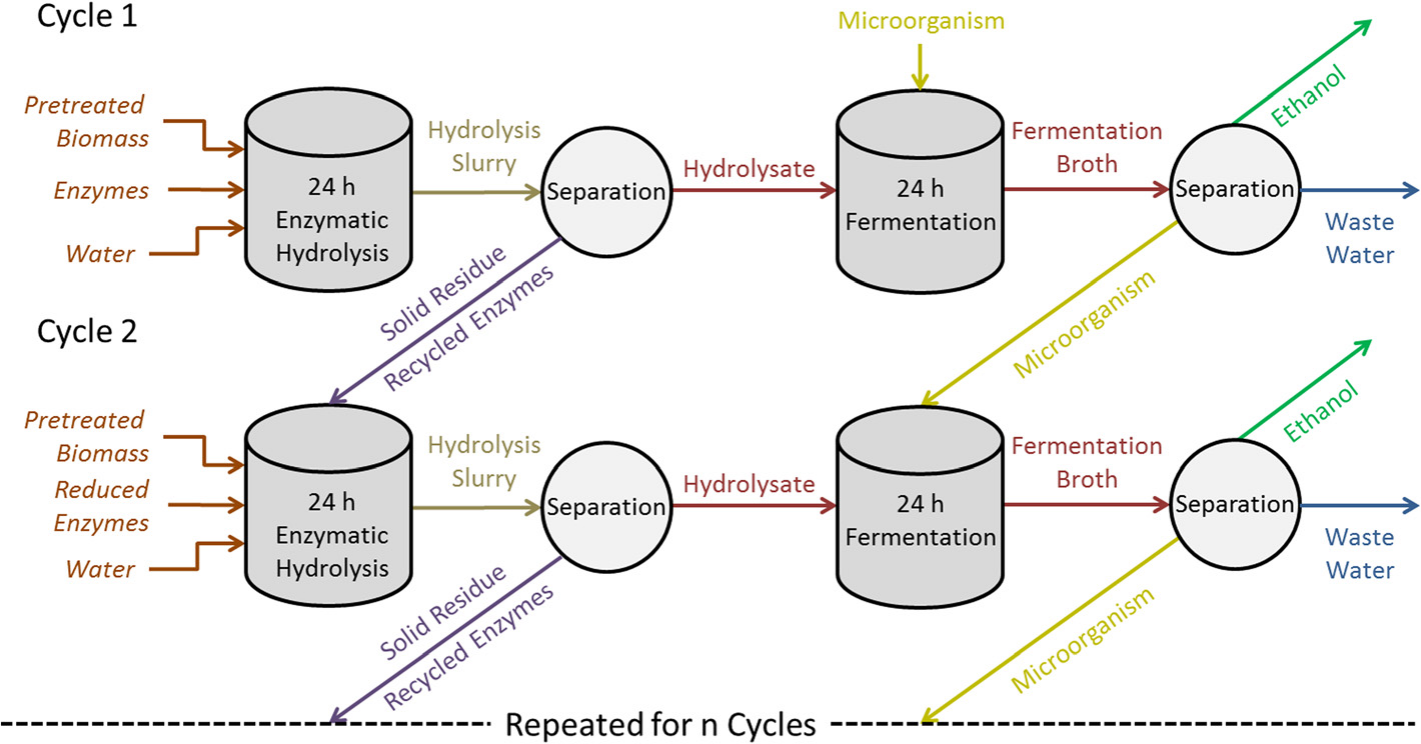
RaBIT process diagram.

The RaBIT process (Figure 1) reduces capital costs by shortening enzymatic hydrolysis and fermentation time to a total of 48 h and can potentially reduce enzyme usage up to 50%. This is accomplished by recycling the unhydrolyzed solids and the accompanying adsorbed enzymes into the next cycle of enzymatic hydrolysis. This will lead to a buildup of unhydrolyzed solids. For a large number of cycles, a portion of the unhydrolyzed solids will need to be removed periodically. Future research is needed to address this issue. To overcome slow xylose utilization, long fermentation times, and low ethanol productivity, the fermentation rate is increased by utilizing a high initial cell density (approximately 10 g/L dry cell weight [DCW]) coupled with cell recycling into the next fermentation stage. As a result, fermentations that normally take 96 to 168 h are completed in 24 h. The use of Ammonia Fiber Expansion (AFEX™) pretreated biomass is highly suitable for this process. AFEX material produces fewer inhibitory compounds compared to dilute acid pretreatment, thereby permitting faster fermentation and providing a high level of viable cells for subsequent fermentation cycles [17]. In addition, AFEX allows pretreated biomass to retain most of its inherent nutrients while adding residual ammonia as a nitrogen source, thereby potentially promoting microbial growth after recycling in the RaBIT process [18]. When utilizing the *Saccharomyces cerevisiae* 424A(LNH-ST) strain in previous research, ethanol titers reached 40 g/L during each cycle and progressively increased. However, the xylose consumption decreased during subsequent cycles despite increasing cell mass [16].

While promising, the feasibility of RaBIT as an industrial process still faces some questions, including the economics of the process, large-scale yeast cell recycling, and high solids operation. However, most of these questions have been answered. Preliminary economic analysis reported by Jin in 2012 showed that the RaBIT process with five-recycle events saved 62% of capital costs associated with hydrolysis and fermentation, had similar centrifugation and filtration costs, and reduced the enzyme cost by 38% [16]. In regard to cell recycling, large-scale cell recycling is commonly performed in the brewing industry and has also been used for fuel ethanol production [19]. High solid loading enzymatic hydrolysis may raise the most questions. However, work towards high solid loading enzymatic hydrolysis is promising, as Jørgensen has shown that a 40% initial dry matter enzymatic hydrolysis is possible when using a horizontal reactor [20]. These results support the industrial capability of the RaBIT process. Future work will need to be performed to confirm these results.

The present work expands on the first version of RaBIT published in 2012 under the BCRL SHF name by investigating high density cell recycling in fermentations [16]. In this work, multiple engineered ethanologens were tested to determine their suitability for the RaBIT process. Three different nutrient sources (corn steep liquor, yeast extract, and wheat germ) were investigated to improve xylose consumption by recycled cells. We also evaluated the potential relationship between cell viability and reduced xylose consumption.

## Results and discussion

### Strain evaluation

Nine different strains were tested for their suitability in high cell density fermentations with cell recycling. Four *Saccharomyces cerevisiae* strains, three *Scheffersomyces stipitis* strains, one *Escherichia coli* strain, and one *Zymomonas mobilis* strain were chosen to represent all major ethanologens available for commercial use. The first goal of our study was to identify a suitable strain to further investigate high cell density fermentations with cell recycle for the RaBIT process. The second goal was to determine if the RaBIT process could be carried out by all ethanologens. Similar fermentation processes have been carried out previously with success by Jin and Fan using *S. cerevisiae* and *Pichia guilliermondii,* respectively [16,21].

**Figure 2:**
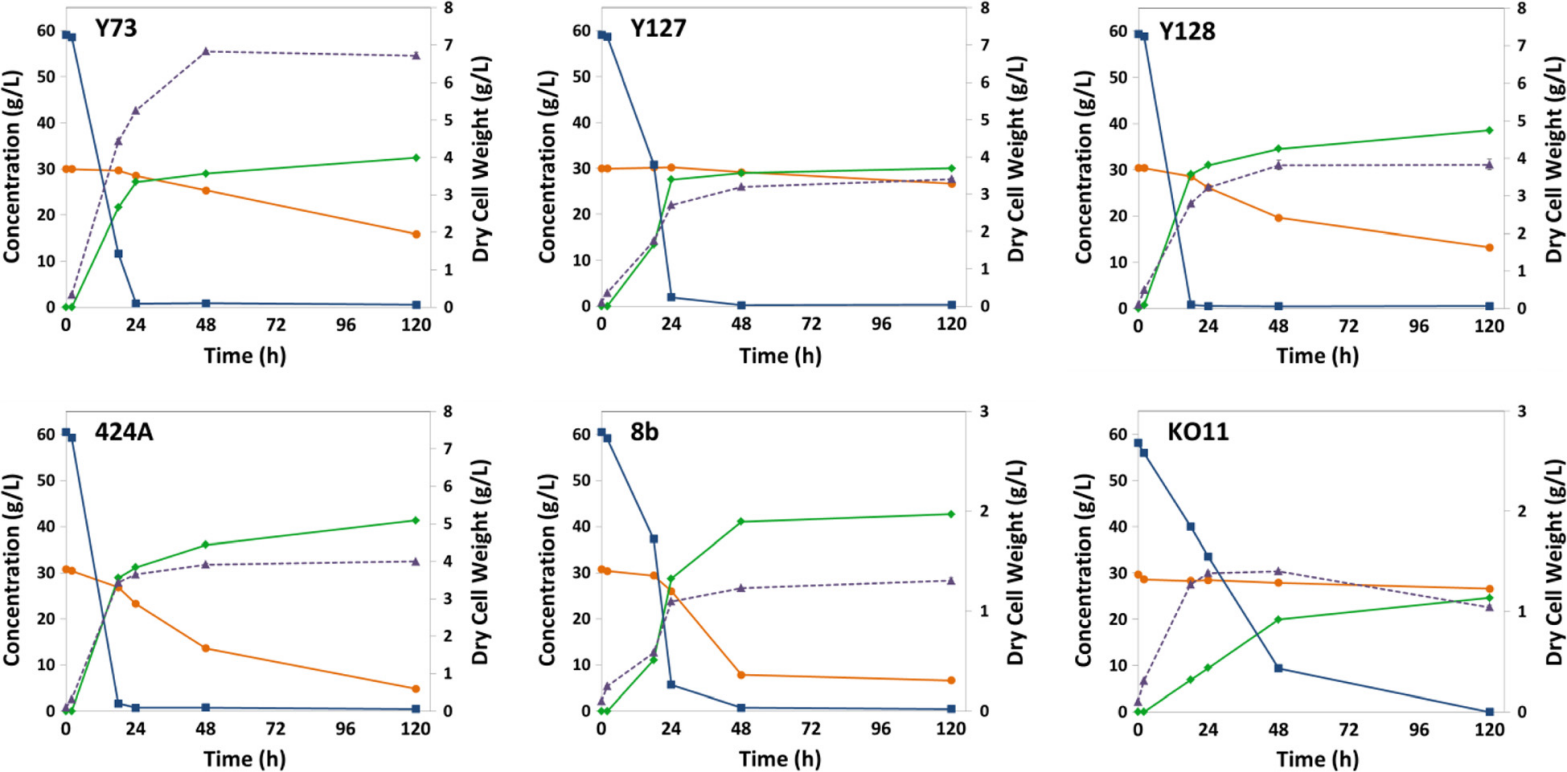
**Strain evaluations during traditional fermentations using AFEX corn stover hydrolysate.** Concentrations are shown for glucose (blue squares), xylose (orange circles), ethanol (green diamonds), and dry cell weight (purple triangles). Error bars are present for all data points, but may be hidden by the symbol.

**Figure 3:**
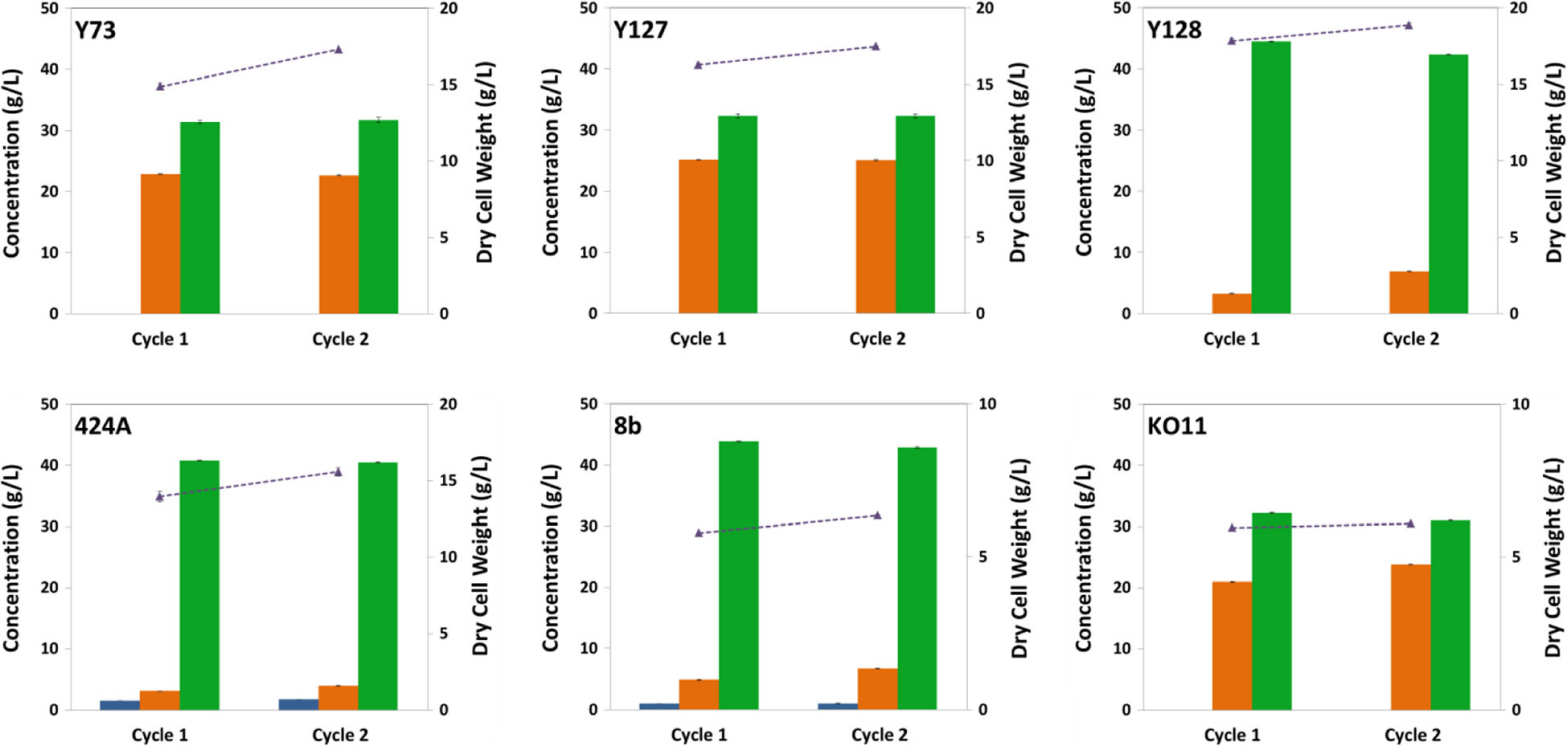
**Strain evaluations during RaBIT fermentations using AFEX corn stover hydrolysate.** The initial glucose and xylose concentrations were 62 g/L and 32 g/L respectively. Final concentrations are shown for glucose (blue), xylose (orange), ethanol (green), and dry cell weight (purple triangles). Error bars are present for all data points, but may be hidden by the symbol.

Strain evaluation was performed using 6% (w/w) glucan loading AFEX treated corn stover hydrolysate. Both traditional fermentations (Figure 2) and RaBIT fermentations (Figure 3) were performed using each strain. By performing both types of fermentations, we hoped to observe correlations between the two processes that would help identify strains suitable for the RaBIT process. In the strain evaluation using traditional fermentation methods, *S. cerevisiae* 424A and *Z. mobilis* 8b showed the best performance, yielding more than 40 g/L ethanol and consuming all but 5 g/L and 6.5 g/L xylose, respectively. Strain 8b was able to consume 75% of the xylose after 48 h, while 424A had only consumed 56% of the xylose by 48 h. *S. cerevisiae* GLBRCY128 (Y128) was the next highest performing strain, yielding 39 g/L ethanol and consuming all but 13 g/L xylose. However, its fermentative rate was much slower than those of 424A and 8b (Table 1). The results summarized in Figure 3 show that three of the nine strains were suitable for RaBIT fermentations: Y128, 424A, and 8b. These three strains were capable of consuming almost all of the glucose and xylose in the first fermentation cycle and produced more than 40 g/L of ethanol. Of the three strains, 424A showed the best potential for recycle due to greater xylose consumption in the second cycle coupled with less reduction in ethanol production during the second cycle. However, Y128 and 8b gave greater ethanol yields. Due to higher xylitol and glycerol production by 424A (data not shown), we hypothesized that use of the xylose isomerase pathway instead of the xylose reductase-xylitol dehydrogenase pathway was a factor for the higher ethanol production per gram of sugar consumed observed with Y128 and 8b [22]. The higher cell mass concentration was seen as a benefit for the 424A and Y128 strains. Excess cell mass can perhaps become a co-product, for example, as animal feed. For these reasons, Y128 was chosen over 424A as the most promising of these nine strains for further evaluation in the RaBIT process. A major goal of these further studies is to understand and then overcome the reduced performance after cell recycling.

**Table 1:** Traditional and RaBIT fermentation comparison

	**Traditional ferm. specific xylose cons. rate**^**+,a**^**, g/g/h**	48 h traditional fermentation EtOH prod.^*,b^**, g/L/h**	120 h traditional fermentation EtOH prod.^*,c^**, g/L/h**	Average RaBIT fermentation EtOH prod.^*,d^**, g/L/h**	**Traditional fermentation EtOH conc., g/L**	**Average RaBIT fermentation EtOH conc., g/L**
Strain						
Y73	0.022 ± 0.001	0.604 ± 0.001	0.271 ± 0.001	1.315 ± 0.017	32.5 ± 0.1	31.6 ± 0.4
Y127	0.015 ± 0.003	0.603 ± 0.007	0.250 ± 0.001	1.346 ± 0.014	30.0 ± 0.2	32.3 ± 0.3
Y128	0.077 ± 0.003	0.720 ± 0.006	0.322 ± 0.001	1.808 ± 0.045	38.6 ± 0.1	43.4 ± 1.1
424A	0.107 ± 0.001	0.752 ± 0.002	0.345 ± 0.003	1.694 ± 0.005	41.3 ± 0.3	40.7 ± 0.1
8b	0.650 ± 0.011	0.856 ± 0.004	0.356 ± 0.001	1.808 ± 0.021	42.7 ± 0.01	43.4 ± 0.5
KO11	0.018 ± 0.003	0.416 ± 0.001	0.205 ± 0.000	1.319 ± 0.026	24.6 ± 0.0	31.6 ± 0.6

^+^Specific xylose consumption rate was calculated by dividing the xylose consumed by the time period and average dry cell weight concentration as correlated from OD measurements; ^*^ethanol productivity was calculated by dividing the ethanol concentration by time of fermentation;

calculated from ^a^24 to 48 h, ^b^0 to 48 h, ^c^0 to 120 h, or ^d^0 to 24 h;

average RaBIT fermentation calculations were performed by averaging the data from the two cycles.

*E. coli* KO11 performance was vastly improved in the RaBIT fermentation compared to the traditional fermentation. *E. coli* KO11 was able to consume almost three times as much xylose and produce more than 7.5 g/L more ethanol in the 24-h RaBIT fermentation compared to the 120-h traditional fermentation. Interestingly, Y128 also showed a large improvement by consuming 8 g/L more xylose, while producing 5 g/L more ethanol in the RaBIT fermentation compared to the traditional 120-h fermentation. The four other strains studied gave comparable performance in the two (traditional and RaBIT) fermentation processes.

Results for the *S. stipitis* strains were not shown in Figures 2 and 3. *S. stipitis* FPL-061 performed comparably to *S. cerevisiae* GLBRCY73 (Additional file 1 and Additional file 2). However, *S. stipitis* FPL-DX26 and Y-7124 were not capable of consuming most of the glucose during RaBIT fermentations (Additional file 2). We hypothesize that the latter two strains require supplemental oxygen as typical for most *S. stipitis* strains. In our view, supplementation of oxygen would not be economically practical for industrialization of the RaBIT process. For those reasons, we chose to exclude the results for the *S. stipitis* strains from comparisons in Figures 2 and 3, as they do not fully represent the potential of *S. stipitis* under these experimental conditions.

Comparing the results of the traditional and RaBIT fermentations, the performance of the RaBIT process seems to be tied to the specific xylose consumption rate. The three strains (424A, 8b, and Y128) with a specific xylose consumption rate greater than 0.075 g/g/h were capable of performing RaBIT process fermentations (Table 1). An assumption is that all strains have a cell population ceiling that depends on the availability of sugar and nutrients. The ceiling in the RaBIT fermentation system depends on cell maintenance needs, cell biomass yields on substrates, and cell growth/death rate. The cell population ceiling is the maximum cell density that could be sustainably maintained in a RaBIT fermentation system. It would then be necessary for each strain to have a sufficient specific xylose consumption rate to consume the xylose in 24 h when near or below this ceiling. An initial cell density above the ceiling results in improved performance during the first cycle, but poor performance after recycling of the cells (data not shown). For the typical *S. cerevisiae* strains, the required xylose consumption rate appears to be around 0.075 g/g/h.

Overall, this comparison also shows one of the key benefits of the RaBIT process, namely increased ethanol productivity (gram EtOH/fermentation volume/time). The RaBIT fermentations increased ethanol productivity more than twofold for the three suitable strains (Table 1). For Y128 specifically, the ethanol productivity increased by 2.5 times for the RaBIT fermentation compared to a 48-h traditional fermentation.

### Process optimizations

**Figure 4:**
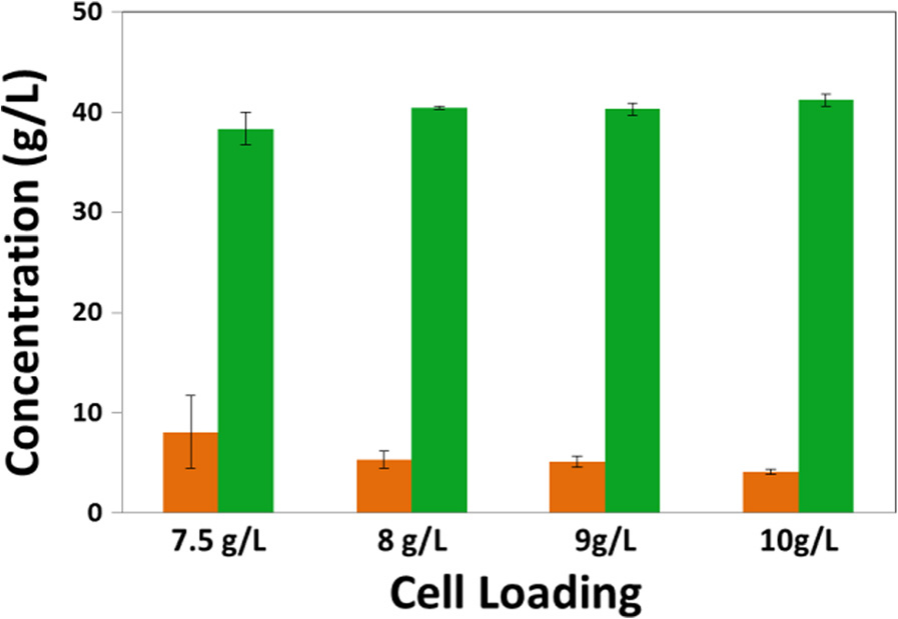
**Effect of different initial cell loadings during RaBIT fermentation.** Final concentrations are shown for xylose (orange) and ethanol (green). Cell loadings are reported as dry cell weight concentration.

After Y128 was selected for further study, the initial cell loading, initial pH, and temperature for RaBIT fermentations (24 h) were optimized. Initial cell loading is the key to rapid fermentation and was examined in 6.0% glucan loading hydrolysate. Cell loadings of 10 g/L, 9 g/L, 8 g/L, and 7.5 g/L (DCW) were tested at 30°C and an initial pH of 5.5 (Figure 4). All initial cell loadings were able to perform the RaBIT process effectively. However, only an initial cell loading of 10 g/L DCW was able to consistently achieve our goal of consuming all but <5 g/L xylose.

**Figure 5:**
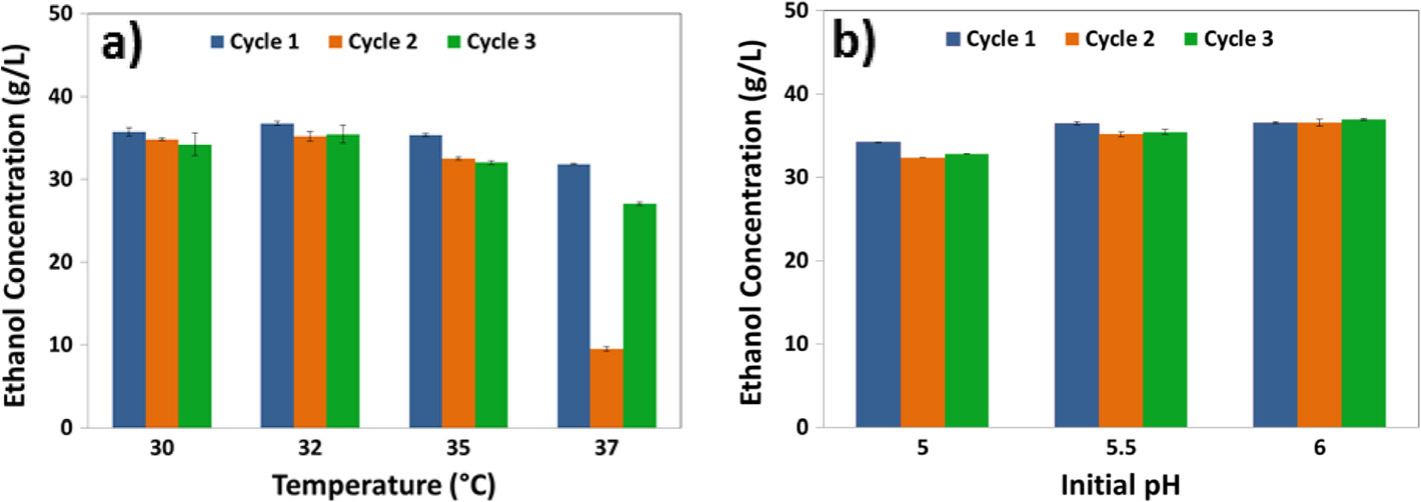
**Optimization of temperature and pH for three-cycle RaBIT fermentation process.** Temperature optimization **(a)** was performed at an initial pH of 5.5 and initial cell loading of 7.5 g/L DCW. pH optimization **(b)** was performed at a temperature of 32°C and initial cell loading of 7.5 g/L DCW. Final ethanol concentrations are shown.

To investigate the effects of temperature and initial pH on the RaBIT fermentation, an initial cell loading of 7.5 g/L DCW was used and three-cycle RaBIT fermentations were performed. Using a cell loading of 7.5 g/L DCW would not be sufficient to consume the desired level of xylose (below 5 g/L at the end of fermentation) allowing clearer observance of improvement in xylose consumption due to changing temperatures and pH. The optimum temperature was determined using an initial pH of 5.5. The results (Figure 5a) showed that increasing the temperature from 30°C to 32°C did not significantly affect the fermentation, with only 1 g/L more ethanol produced on average at 32°C compared to 30°C. Performance decreased at 35°C with 2.5 g/L less ethanol produced on average compared to 32°C. At 35°C, the ethanol metabolic yield was possibly reduced due to maintenance requirements. The fermentations performed at 37°C greatly affected the cell population. The 70% drop in ethanol production during cycle 2 appeared to be due to significant cell death at the elevated temperature. At the end of the first cycle, no viable colonies were found when plating at 50^4^ times dilution. Viable colonies were found for all other temperatures and cycles. Also, optical density (OD) measurements indicated that the cell mass at the end of cycles 1 and 2 was less than that of the initial inoculum.

The final optimization test determining the optimal initial pH is shown in Figure 5b. At 32°C and 7.5 g/L DCW initial loading, the optimal pH was 6.0. At this pH, the highest ethanol titers were reached. Furthermore, for the first time during this work, ethanol production increased after both recycling events. An initial pH of 6.5 was also attempted, but produced unstable cell behavior as manifested by the large variability in results (data not shown). Another experiment was performed to determine if the higher pH was beneficial due to the physiological state of the cell or due to precipitation of inhibiting compounds (data not shown). Hydrolysates prepared by solely raising the pH from 4.8 (enzymatic hydrolysis pH) to 5.0, 5.5, or 6.0 were compared fermentatively to hydrolysates that were raised to pH 6, sterile filtered, and then acidified back down to 5.0, 5.5, or 6.0. The study was necessary as raising pH can cause the removal/precipitation of degradation products as commonly practiced in overliming [23]. The results showed no significant difference in fermentability of the two sets of hydrolysates, indicating that the pH was affecting physiological state rather than precipitating inhibitors.

### Nutrient testing

**Figure 6:**
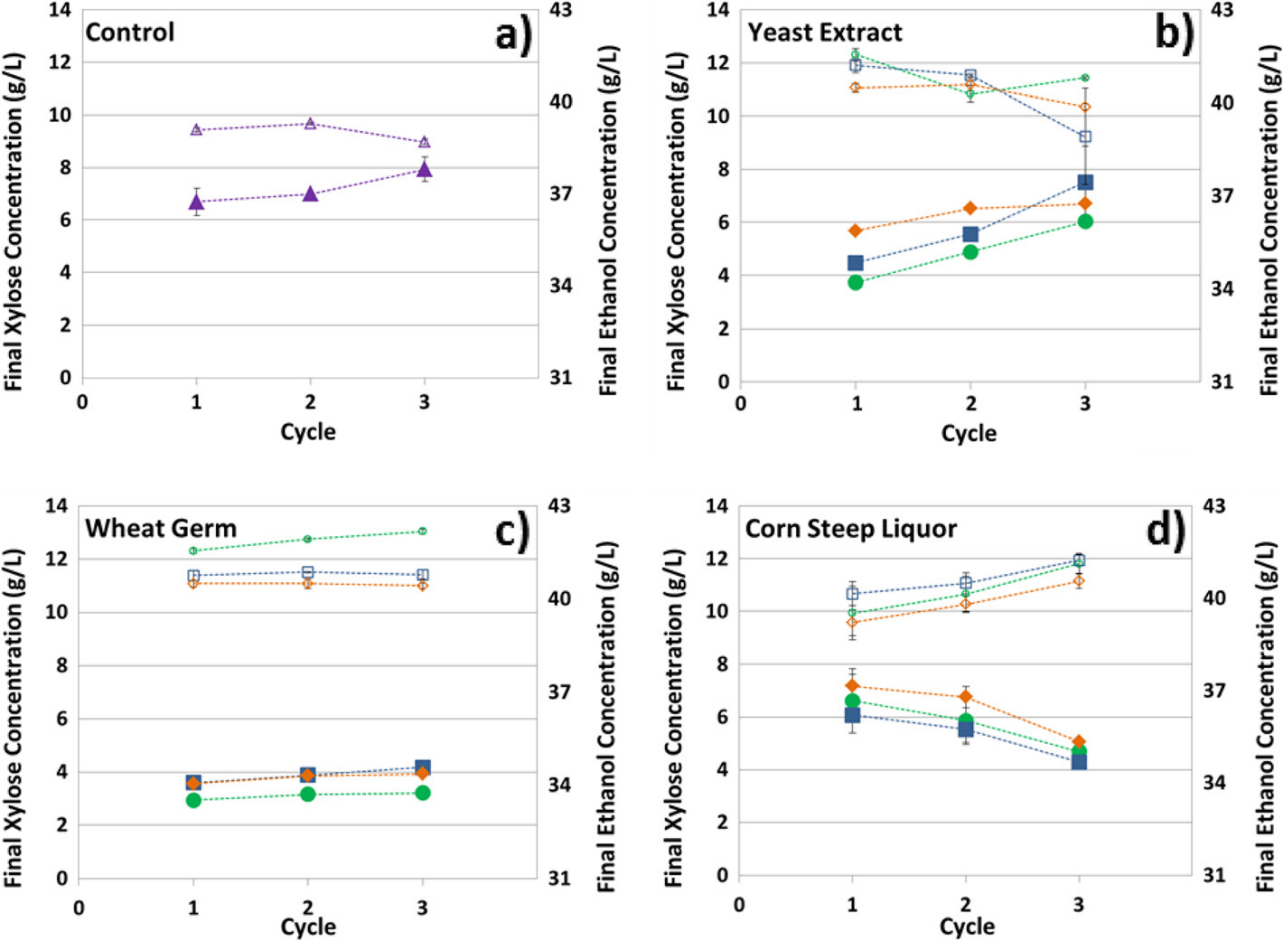
**Effect of nutrient addition on RaBIT fermentation process.** Fermentation conditions consisted of 6% glucan loading hydrolysate, 32°C, initial pH of 6.0, and initial cell loading of 7.5 g/L DCW. Closed symbols represent xylose concentration; open symbols represent ethanol concentration. Tested were **a)** no nutrients or nutrient concentrations of 1 g/L (orange diamonds), 2.5 g/L (blue squares), and 5.0 g/L (green circles) using **b)** yeast extract, **c)** wheat germ, or **d)** corn steep liquor. Initial glucose and xylose concentrations were approximately 58 g/L and 29 g/L, respectively. Error bars are present for all data points, but may be hidden by the symbols.

As shown in Figure 3, xylose consumption decreases upon recycling Y128. Decreasing xylose consumption was also experienced during the optimization work (data not shown). Lack of sufficient nutrients may be one reason for decreasing xylose consumption through additional cycles. AFEX-treated corn stover supports cell growth to high concentrations [17]. However, there may not be enough nutrients present to fully support the high cell populations in the demanding RaBIT process conditions. Three different nutrient sources were tested: yeast extract, wheat germ, and corn steep liquor. Yeast extract, the product of autolysed yeast cells, was used as a potentially ideal nutrient source. However, industrially yeast extract would probably not be feasible due to its high price. Corn steep liquor (CSL) and wheat germ were chosen as cheaper and more practical options. CSL is the cheaper of the two and is produced as a by-product of corn wet-milling [24]. CSL provides a reasonable amount of nutrients, but also contains inhibitors such as lactic acid [24]. Furthermore, CSL is well established as a nutrient source for industrial fermentations [25]. Wheat germ is a by-product of flour milling [26]. It contains high levels of metals such as zinc and magnesium (Additional file 3), which have been shown to help yeast resist ethanol stress [27-29]. The addition of yeast extract (Figure 6b) did not benefit the fermentation very much. Compared to control experiments, the addition of up to 5 g/L yeast extract improved the xylose consumption by about 2 g/L and showed up to 2 g/L higher ethanol production. However, yeast extract addition did not prevent decreased xylose consumption.

Wheat germ was added during the enzymatic hydrolysis to help release the nutrients (Figure 6c). The addition of 5.0 g/L wheat germ improved the overall xylose consumption by up to 3.5 g/L and ethanol production by up to 4.5 g/L for the third cycle. These results concur with our initial hypothesis that wheat germ would allow the yeast to resist the higher ethanol concentrations by consuming more xylose and lowering the cell maintenance energy requirements. The xylose consumption, however, still decreased during subsequent cycles.

The addition of CSL to the fermentation broth gave the best results (Figure 6d). CSL promoted increased xylose consumption in subsequent cycles. This was observed at concentrations of 1, 2.5, and 5 g/L. The best results were at 2.5 g/L. At the higher concentration of 5 g/L, ethanol production decreases, likely due to excess cell growth or inhibition from the CSL. In the third cycle, the addition of CSL caused 3.5 g/L more xylose consumption and 2.5 g/L more ethanol production compared to the control. Additionally, the improvement between the first and third cycles showed 2 g/L more consumed xylose and 1.25 g/L more ethanol. This may indicate an increase in cell viability across cycles compared to the drop seen in all other cases.

Wheat germ and CSL were also added in combination (Additional file 4). We expected better ethanol production with increased xylose consumption after each cycle. However, the results were similar to those for yeast extract addition. There was an initial benefit to the fermentation but still a drastic decrease in xylose consumption and ethanol production as the cycles progressed.

The final nutrient test was performed by adding CSL (2.5 g/L) during the xylose consumption phase (at 6 h) rather than at the beginning of the fermentation (Additional file 5). In the end, the addition of the CSL at the beginning was more beneficial to both xylose consumption and ethanol production. This indicates that nutrient addition is more important during the high growth phase than during high stress phase. However, the xylose consumption still improved over each cycle regardless of when the CSL was added.

### Five-cycle viable cell profiling

The five-cycle comparisons were performed in a bioreactor to better imitate industrial conditions. The experimental goal was to profile the viable cell mass through five cycles with the use of a capacitance probe. A no nutrient addition case was compared to the optimal nutrient addition case (2.5 g/L CSL) as determined previously. The five-cycle comparison used the optimal initial inoculum of 10 g/L DCW, initial pH of 6.0, and 32°C. Previously, cell population was measured using the OD method, which does not accurately measure the viable cell population. Traditionally, viability plating or staining can be used to measure cells capable of growth or cells with intact cellular membranes, respectively. Because the Y128 strain flocculates, these traditional methods were not sufficiently accurate. This problem was solved using a capacitance probe. Cells with intact membranes give a capacitance reading when an electrical current is passed around them. When the membrane is compromised, the current can pass through the cells, and this capacitance is lost. Thus, capacitance readings can measure cell biomass with intact membranes, while not including cells with disrupted membranes [30,31]. Capacitance readings were taken every 10 seconds and averaged over 10 readings to reduce signal noise. An accurate viable cell profile was necessary for determining the cause of reduced xylose consumption as the cycles increased. From previous OD measurements, there appeared to be only minor or no growth after the first cycle. A lack of growth or death could create a cell population that is accumulating biomass degradation products inside the cell causing reduced metabolic activity. Furthermore, the OD measurements may not have accurately measured cell death. The outer membranes of some cells may have been disrupted enough to stop metabolic activity, but still have enough integrity to scatter the light associated with an OD measurement. Accurate viable cell measurements would also help determine if CSL addition benefited cell growth or cell metabolism.

**Figure 7:**
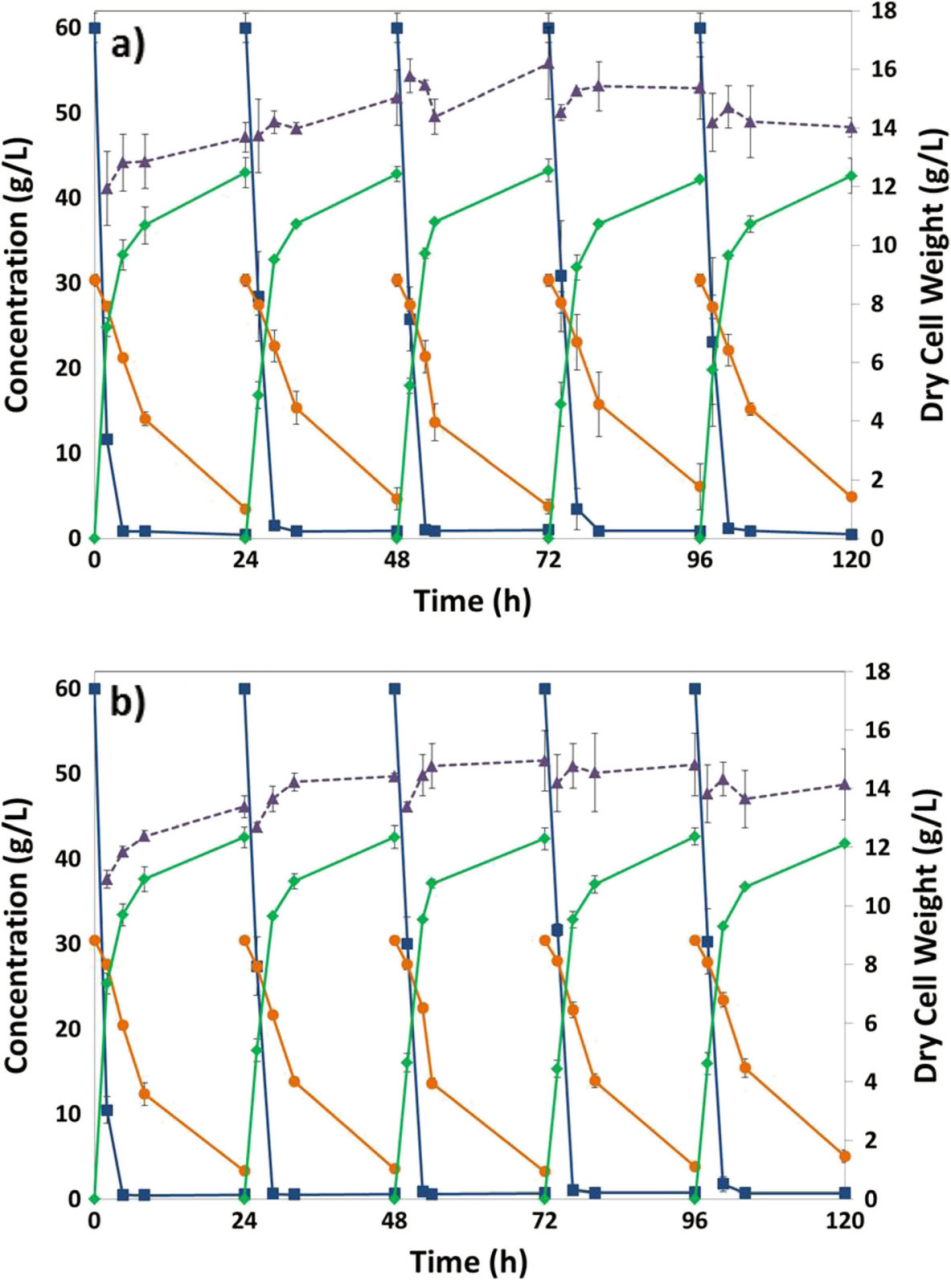
**RaBIT fermentation process comparison in the presence and absence of nutrient supplementation.** Here, **a)** shows results for no nutrient addition and **b)** those for 2.5 g/L CSL addition. Concentrations are shown for glucose (blue squares), xylose (orange circles), ethanol (green diamonds), and dry cell weight correlated from OD (purple triangles).

The sugar, ethanol, and OD measurements are given in Figure 7. Overall, 2.5 g/L added CSL slightly improved the performance compared to no CSL addition with regard to xylose consumption. With CSL addition, final xylose concentrations were 3.5 ± 0.25 for the first four cycles. Without the CSL addition, final xylose concentrations were 3.5 g/L, 4.7 g/L, 3.8 g/L, and 6.1 g/L for cycles 1 through 4, respectively. Cycle 5 xylose concentrations and cycles 1 through 5 ethanol concentrations were comparable for the two cases.

**Figure 8:**
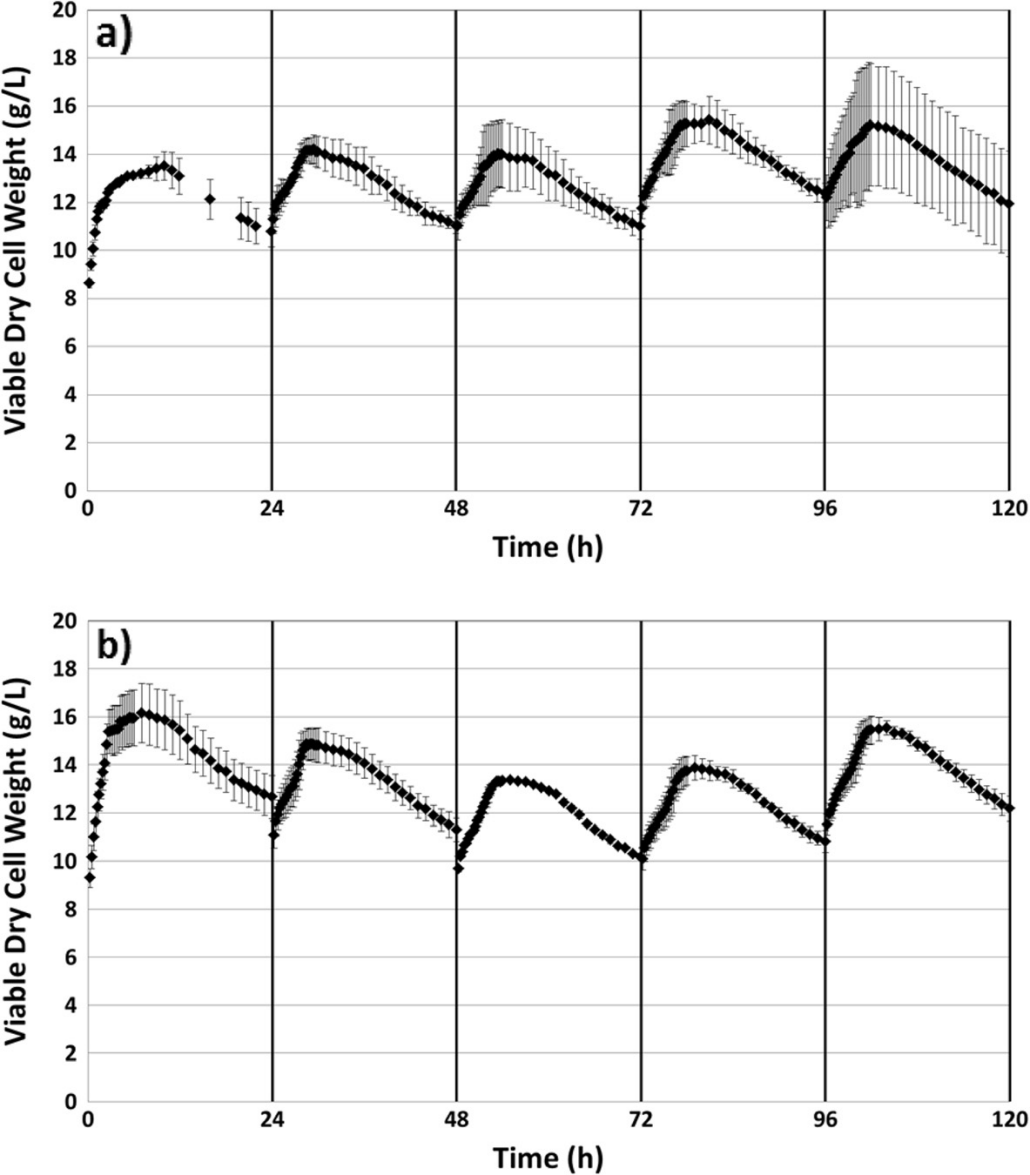
**Measure of viable dry cell weight.** Viable DCW was correlated from capacitance reading for five-cycle RaBIT fermentations with **a)** no added nutrients and **b)** 2.5 g/L added corn steep liquor.

Figure 8 shows the viable cell density profile for both no nutrient and 2.5 g/L added CSL through five cycles. For all cycles, the viable cell density increases during the first 5 to 7 h. The growth phase appears to end shortly after all the glucose is consumed. After a brief stationary phase, the viable cell density then decreases rapidly during the rest of the xylose consumption phase. This general trend was observed during all cycles and for both cases: no nutrients added and 2.5 g/L added CSL.

There was a large difference in the fermentation kinetics between the two cases (Table 2). Overall, added CSL showed faster growth rates and faster death rates. Interestingly, when no nutrients were added, the death rate increased in later cycles. When CSL was added, no dramatic variations in death rate were observed. Differences were also present in the specific xylose consumption rates (gram xylose consumed/gram viable cell mass/hour). When no nutrients were added, the specific xylose consumption rate was lower during the last four cycles compared to the first cycle. When CSL was added, the specific xylose consumption rate was higher during cycles 2 through 4 compared to cycle 1. This indicated that cell populations were more metabolically active with the addition of CSL.

**Table 2:** RaBIT fermentation cellular rates

	**Specific xylose cons. rate**^**+,a**^**, g/g/h**	**Average viable cell density**^**a**^**, g/L DCW**	**Growth rate**^**b**^**, g/L/h**	**Death rate**^**c**^**, g/L/h**
No nutrients				
Cycle 1	0.092 ± 0.005	12.3 ± 0.5	0.401 ± 0.113	-0.156 ± 0.024
Cycle 2	0.084 ± 0.001	12.8 ± 0.5	0.624 ± 0.356	-0.176 ± 0.028
Cycle 3	0.088 ± 0.006	12.7 ± 0.9	0.547 ± 0.383	-0.176 ± 0.050
Cycle 4	0.072 ± 0.003	14.0 ± 0.7	0.579 ± 0.326	-0.180 ± 0.020
Cycle 5	0.079 ± 0.014	13.8 ± 2.0	0.478 ± 0.315	-0.197 ± 0.019
2.5 g/L CSL				
Cycle 1	0.079 ± 0.003	14.4 ± 1.0	0.831 ± 0.158	-0.214 ± 0.024
Cycle 2	0.084 ± 0.006	13.3 ± 0.6	0.841 ± 0.056	-0.208 ± 0.012
Cycle 3	0.094 ± 0.001	12.0 ± 0.0	0.728 ± 0.070	-0.196 ± 0.005
Cycle 4	0.089 ± 0.003	12.5 ± 0.4	0.718 ± 0.009	-0.189 ± 0.002
Cycle 5	0.076 ± 0.001	14.0 ± 0.4	0.688 ± 0.162	-0.210 ± 0.015

^+^Specific xylose consumption rate was calculated by dividing the xylose consumed by the time period and average viable dry cell weight concentration as correlated from capacitance readings;

calculated from ^a^0 to 24 h, ^b^2 to 4.5 h, or ^c^8 to 24 h;

average cell viable cell concentration was calculated using the integral method.

## Conclusion

We found that not all ethanologens are suitable for RaBIT platform fermentations. Of the nine tested ethanologens, *Saccharomyces cerevisiae* 424A(LNH-ST), *Zymomonas mobilis* 8b, and *S. cerevisiae* GLBRCY128 showed good performance in the RaBIT fermentation process. Y128 was chosen for optimization of process conditions. Different nutrient supplementation protocols were evaluated to see whether xylose consumption could be improved during subsequent cycles of the RaBIT process. We found that adding 2.5 g/L corn steep liquor (CSL) improved xylose consumption for the three cycles tested when 7.5 g/L initial dry cell weight (DCW) inoculum was used. However, the xylose consumption problems still existed when 10 g/L DCW inoculum was utilized for optimal ethanol production for five fermentation cycles. Capacitance monitoring indicated that there is both dynamic cell growth and death during each RaBIT cycle. Furthermore, the main cause of reduced xylose consumption with subsequent cycles is decreased specific xylose consumption rate rather than decreased viable cell mass.

## Materials and methods

### Biomass and pretreatment

Corn stover was provided by the Great Lakes Bioenergy Research Center (GLBRC). The corn (Pioneer 36H56) from which the stover was produced was planted in May of 2009 in field 570-N at the Arlington Agricultural Research Station in Columbia Country, WI and harvested in November of 2009. The biomass was pretreated by the Biomass Conversion Research Laboratory (BCRL) located at Michigan State University in East Lansing, MI using the AFEX pretreatment process as previously described in the literature [32]. The AFEX pretreatment conditions were 1:1 ammonia to biomass ratio, 60% moisture on dry weight basis, 100°C, and 30 min. reaction time. Glucan, xylan, and acid insoluble lignin content plus ash were 38.0%, 23.8%, and 20.4% by dry mass, respectively. The corn stover was stored at 4°C.

### Microorganisms and seed culture preparation

*Saccharomyces cerevisiae* GLBRCY73 was genetically modified to contain xylose reductase, xylitol dehydrogenase, and xylulokinase genes [33]. *S. cerevisiae strains* GLBRCY127 and GLBRCY128 were genetically modified to contain xylose isomerase and xululokinase genes (T.K. Sato, manuscript in preparation). 424A(LNH-ST) was provided by Prof. Nancy W.H. Ho of Purdue University in West Lafayette, IN. *S. cerevisiae* 424A was genetically modified with multiple copies of xylose reductase and xylitol dehydrogenase genes from *Scheffersomyces (Pichia) stipitis* and an endogenous xylulokinase gene incorporated in the chromosome [34].

*Zymomonas mobilis* 8b was provided by MBI, International (Lansing, MI) and was originally obtained from the National Renewable Energy Laboratory (Golden, CO) [35].

*Scheffersomyces (Pichia) stipitis* FPL-061 and FPL-DX26 strains were provided by Prof. Thomas W. Jeffries of the University of Wisconsin in Madison, WI [36]. NRRL Y-7124 was obtained from ARS Culture Collection (National Center Agricultural Utilization Research, Peoria, IL) [37].

*Escherichia coli* KO11 was obtained from the American Type Culture Collection having designated number 55124 [38].

All strains were maintained in glycerol stocks at -80°C. Seed cultures were prepared in a medium containing 100 g/L dextrose, 25 g/L xylose, 10 g/L yeast extract, and 20 g/L tryptone. Seed cultures were performed in 250 mL Erlenmeyer flasks using a 100 mL working volume. The initial OD_600_ of seed cultures was 0.1. Cultures were incubated at 30°C and 150 RPM for 20 h. After 20 h, 1 mL of the culture was transferred to new media for an additional 20 h. The cultivation was made aerobic by use of a foam stopper for *S. stipitis* strains. All other seed cultures were microaerobic by use of a rubber stopper pierced by a needle.

### Enzymatic hydrolysis

Enzymatic hydrolysis at 6% (w/w) glucan loading was performed in 1 L baffled Erlenmeyer flasks with a reaction mixture of 400 g. Biomass was loaded in fed-batch mode by adding half the biomass at t = 0 h and the other half at t = 2 h. The enzyme cocktail consisted of 20 mg protein/g glucan Cellic CTec2 (Novozymes), 5 mg/g Cellic HTec2 (Novozymes), and 5 mg/g Multifect Pectinase (Genencor). Hydrolysis was performed for 48 h at 50°C and 250 RPM using a pH of 4.8. Adjustments to the pH were made using 10 M potassium hydroxide or 12.1 M hydrochloric acid. Hydrolysis slurry was centrifuged in 2 L bottles at 7,500 RPM for 30 min and then sterile filtered. Hydrolysate was used for fermentation without external nutrient supplementation unless otherwise indicated.

### Fermentations

Fermentations were performed in 125 mL Erlenmeyer flasks using 50 mL of hydrolysate. Cells for inoculation were harvested by centrifugation from the seed cultures. Inoculation size was determined by dry cell weight (DCW) concentration. Inoculations were performed at 0.1 g/L for traditional fermentations, 4 g/L DCW for RaBIT fermentations using *Z. mobilis* and *E. coli,* and 7.5, 8.0, 9.0, 10, or 12.0 g/L DCW for RaBIT fermentations using *S. cerevisiae* and *S. stipitis*. The pH was initially adjusted using 10 M potassium hydroxide. The initial pH for *S. cerevisiae* and *S. stipitis* was 5.5 during strain testing before pH optimization and 6.0 after. The initial pH values for *Z. mobilis* and *E. coli* were 6.0 and 7.0, respectively. The pH for the *E. coli* was buffered using 0.05 M MOPS and adjusted twice daily. The pH for all other strains was not adjusted during the fermentations. The fermentations were performed in a shaking incubator at 150 RPM. The temperature was set at 37°C for *E. coli* and 30°C for all other strains before temperature optimization. After optimization, the temperature was increased to 32°C for *S. cerevisiae* GLBRCY128. The flasks were kept under microaerobic conditions. Traditional fermentations were incubated for five days. RaBIT fermentations were performed for 24 h. At the end of each RaBIT fermentation stage, the broth was centrifuged in 50 mL centrifuge tubes at 4,000 RPM for 10 min. The corresponding cell pellets were then inoculated into fresh hydrolysate to begin the next cycle. All fermentation experiments were performed with at least two biological replicates.

### Five-cycle fermentation in bioreactor

Five-cycle RaBIT fermentations were performed in a 0.5 L bioreactor with a 60% working volume. Temperature and stirring rate were set at 32°C and 300 RPM, respectively. A 6% glucan loading hydrolysate (60 g/L glucose and 30 g/L xylose) with an initial pH of 6.0 and 10 g/L DCW inoculum were used. A capacitance probe was utilized to monitor viable cell density. The recycle process was carried out as described in the Fermentations section above.

### Nutrient additions

In the nutrient addition experiments, yeast extract (Becton Dickinson), corn steep liquor (Sigma Aldrich), and wheat germ (MP Biomedicals) were added at concentrations of 1.0, 2.5, or 5.0 g/L. Yeast extract and corn steep liquor were weighed out and added to the hydrolysate before fermentation. Wheat germ was added to the enzymatic hydrolysis mixture at the beginning of the hydrolysis (the final mixture density was assumed as 1 g/L).

### Measurements of cell population

The optical density at 600 nm was used to measure the cell concentration of the fermentation broths. The OD_600_ measurement was then correlated to the DCW by use of a calibration curve.

Viable cell mass was measured by correlating capacitance reading from an Aber Instruments Ltd. Biomass Monitor 200. The capacitance versus viable dry cell mass correlation was created by taking samples during exponential phase seed cultures. The samples were centrifuged and dried before being compared to the capacitance readings, which produced a linear correlation.

### HPLC analysis

Glucose, xylose, and ethanol concentrations were analyzed by HPLC using a Biorad Aminex HPX-87H column. The column temperature was maintained at 50°C. The mobile phase (5 mM H_2_SO_4_) flow rate was 0.6 mL/min.

## Abbreviations

424A: *Saccharomyces cerevisiae* 424A(LNH-ST) strain; 8b: *Zymomonas mobilis* 8b strain; AFEX: Ammonia Fiber Expansion; BCRL: Biomass Conversion Research Laboratory; CSL: corn steep liquor; DCW: dry cell weight; E. coli: *Escherichia coli*; EtOH: ethanol; GLBRC: Great Lakes Bioenergy Research Center; HPLC: high performance liquid chromatography; OD: optical density; RaBIT: Rapid Bioconversion with Integrated recycle Technology; S. cerevisiae: *Saccharomyces cerevisiae*; SHF: separate hydrolysis and fermentation; SSCF: simultaneous saccharification and co-fermentation; S. stipitis: *Scheffersomyces stipitis*; Y128: *Saccharomyces cerevisiae* GLBRCY128; Z. mobilis: *Zymomonas mobilis.*

## Competing interests

The authors declare that they have no competing interests.

## Authors’ contributions

CS designed and performed the experiments, analyzed the data, and wrote the manuscript. MJ supervised the study, participated in the experimental design, helped in interpreting the results, and edited the manuscript. TS coordinated the experiment, provided experimental direction, and edited the manuscript. VB coordinated the experiment and edited the manuscript. BD supervised and edited the manuscript. All authors read and approved the final manuscript.

## Supplementary Material

Additional file 1: Figure S1 Strain evaluations during traditional fermentations using AFEX corn stover hydrolysate. Concentrations are shown for glucose (blue squares), xylose (orange circles), ethanol (green diamonds), and dry cell weight (purple triangles). Error bars are present for all data points, but may be hidden by marks. Click here for file

Additional file 2: Figure S2 Strain evaluations during RaBIT fermentations using AFEX corn stover hydrolysate. The initial glucose and xylose concentrations were 62 g/L and 32 g/L, respectively. Final concentrations are shown for glucose (blue), xylose (orange), ethanol (green), and dry cell weight (purple triangles). Error bars are present for all data points, but may be hidden by marks. Click here for file

Additional file 3: Table S1 Nutrient additive compositions. Click here for file

Additional file 4: Figure S3 Combination of corn steep liquor and wheat germ at a 50% ratio as a nutrient source. Closed symbols represent xylose concentration; open symbols represent ethanol concentration. Total concentrations of 1 g/L (blue squares) and 2 g/L (green circles) were tested. Click here for file

Additional file 5: Figure S4 2.5 g/L corn steep liquor addition time testing. Closed symbols represent xylose concentration; open symbols represent ethanol concentration. Additions were made at t = 0 h (blue squares) and t = 6 h (green circles). Click here for file
