# Supplementary figures and images for: Studying the rapid bioconversion of lignocellulosic sugars into ethanol using high cell density fermentations with cell recycle
Source: Biotechnol Biofuels. 2014 May 15;7:73. doi: 10.1186/1754-6834-7-73 (PMC4026590; doi:10.1186/1754-6834-7-73)

**
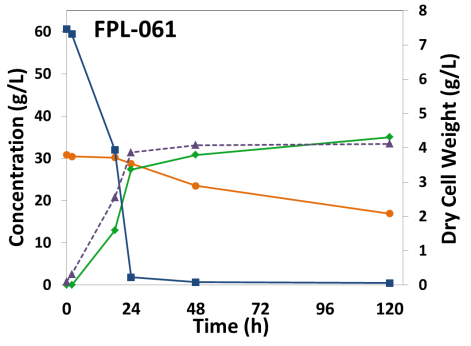

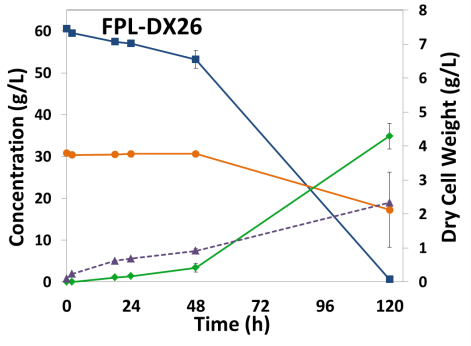

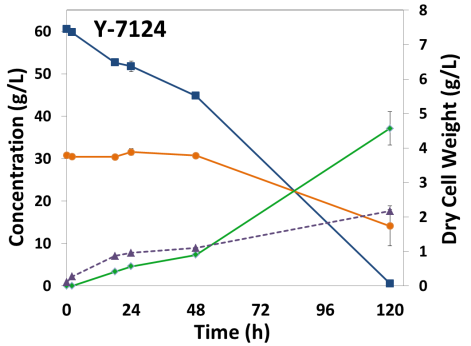
**

Supplement: Additional file 1: Figure S1 — Strain evaluations during traditional fermentations using AFEX corn stover hydrolysate. Concentrations are shown for glucose (blue squares), xylose (orange circles), ethanol (green diamonds), and dry cell weight (purple triangles). Error bars are present for all data points, but may be hidden by marks. [file 1754-6834-7-73-S1.docx]

***
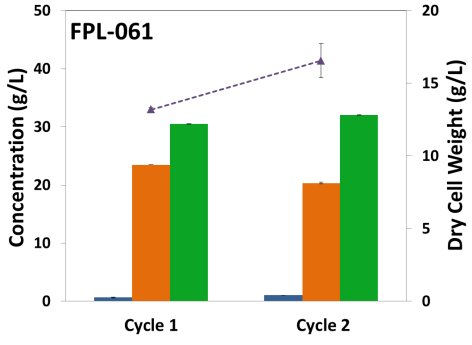

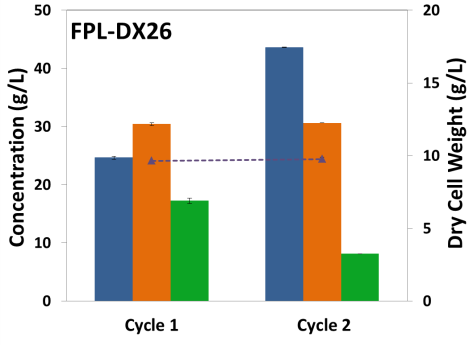

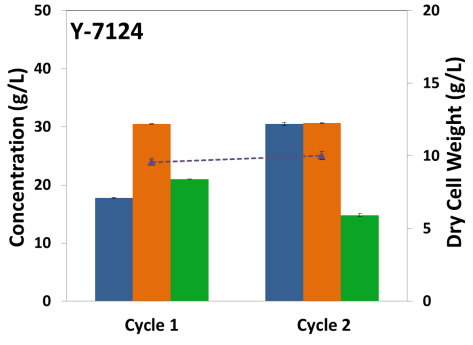
***

Supplement: Additional file 2: Figure S2 — Strain evaluations during RaBIT fermentations using AFEX corn stover hydrolysate. The initial glucose and xylose concentrations were 62 g/L and 32 g/L, respectively. Final concentrations are shown for glucose (blue), xylose (orange), ethanol (green), and dry cell weight (purple triangles). Error bars are present for all data points, but may be hidden by marks. [file 1754-6834-7-73-S2.docx]

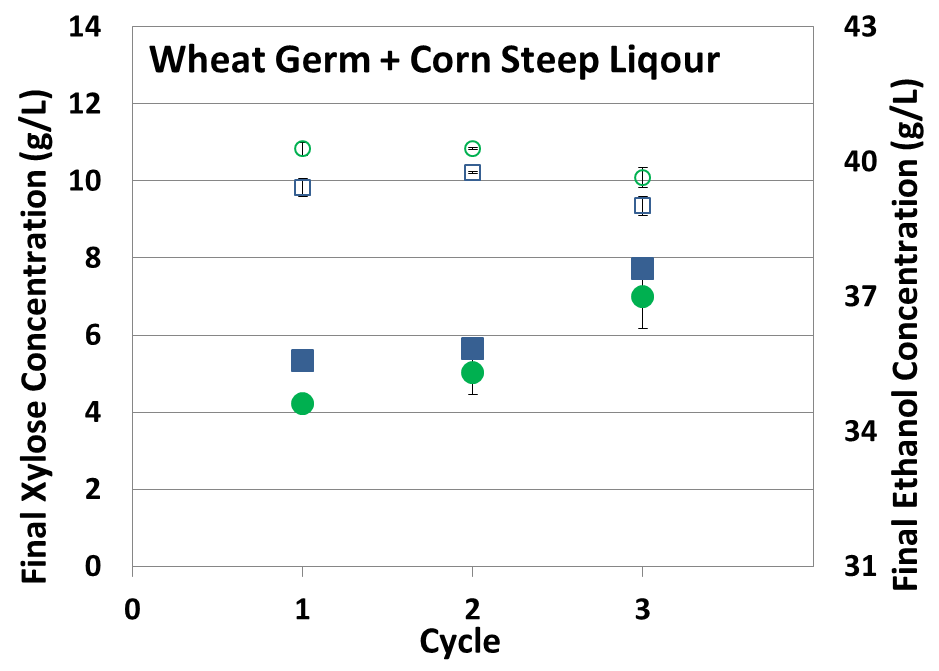

Supplement: Additional file 4: Figure S3 — Combination of corn steep liquor and wheat germ at a 50% ratio as a nutrient source. Closed symbols represent xylose concentration; open symbols represent ethanol concentration. Total concentrations of 1 g/L (blue squares) and 2 g/L (green circles) were tested. [file 1754-6834-7-73-S4.docx]

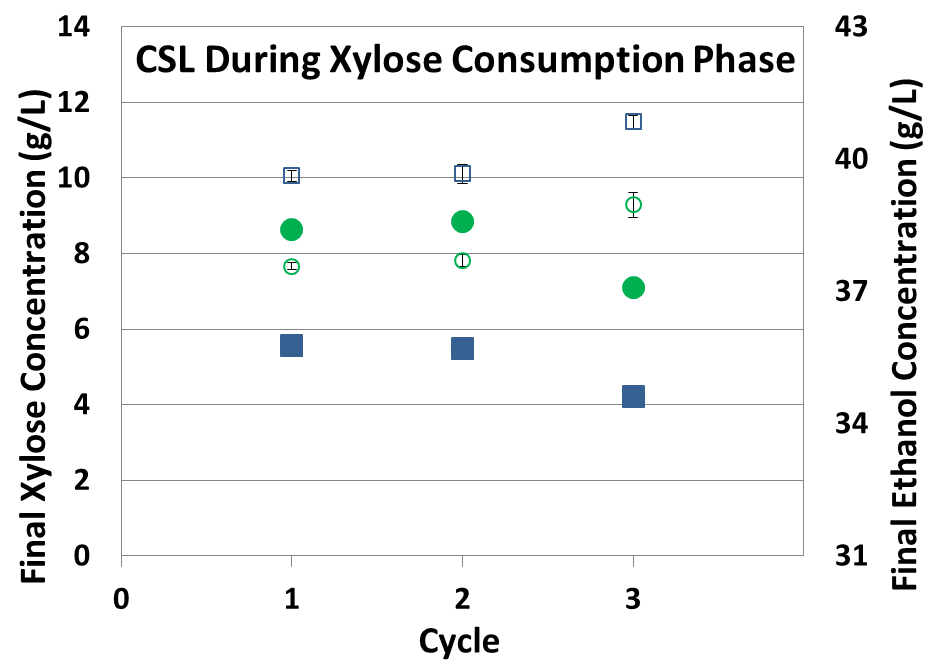

Supplement: Additional file 5: Figure S4 — 2.5 g/L corn steep liquor addition time testing. Closed symbols represent xylose concentration; open symbols represent ethanol concentration. Additions were made at t = 0 h (blue squares) and t = 6 h (green circles). [file 1754-6834-7-73-S5.docx]
