# Supplementary material for: Studying the rapid bioconversion of lignocellulosic sugars into ethanol using high cell density fermentations with cell recycle
Source: Biotechnol Biofuels. 2014 May 15;7:73. doi: 10.1186/1754-6834-7-73 (PMC4026590; doi:10.1186/1754-6834-7-73)
Supplement: Additional file 3: Table S1 — Nutrient additive compositions. [file 1754-6834-7-73-S3.docx]

| **Table S1** Nutrient Additive Compositions | | | |
| --- | --- | --- | --- |
|  | Corn Steep Liquor[21] | Yeast Extract[35] | Wheat Germ[36] |
| Water | 45-50% | 3.10% | 11.12% |
| Total N | 2.7-4.5% | 10.90% | - |
| Amino N | 1-1.8% | 6% | - |
| Ash | 9-10% | 11.20% | - |
| Ca | 0.5-1.5 pdm | 130ug/g | 39ug/g |
| Cu | 0-0.001 pdm | - | 0.79ug/g |
| Fe | 0.01-0.05 pdm | 55.3ug/g | 6.26ug/g |
| Mg | 0.5-1.0 pdm | 750ug/g | 239ug/g |
| Mn | 0.004-0.0125 pdm | - | 13.30ug/g |
| K | 1-25 pdm | 31950ug/g | 842ug/g |
| Na | - | 4900ug/g | 12ug/g |
| P | 2.0-3.0 pdm | - | 892ug/g |
| Phosphate | - | 3.27% | - |
| S | 0.34 pdm | - | - |
| Sulfate | - | 0.09% | - |
| Zn | 0.0005-0.005 pdm | - | 12.29ug/g |
| pdm = percent dry matter | | | |
